# Ginger Loaded Polyethylene Oxide Electrospun Nanomembrane: Rheological and Antimicrobial Attributes

**DOI:** 10.3390/membranes12111148

**Published:** 2022-11-16

**Authors:** Anum Javaid, Mohammed Jalalah, Rimsha Safdar, Zubair Khaliq, Muhammad Bilal Qadir, Sumra Zulfiqar, Adnan Ahmad, Aamir Naseem Satti, Aiman Ali, M. Faisal, S. A. Alsareii, Farid A. Harraz

**Affiliations:** 1 Department of Materials, National Textile University, Faisalabad 37610, Pakistan; 2 Promising Centre for Sensors and Electronic Devices (PCSED), Advanced Materials and Nano-Research Centre, Najran University, P.O. Box 1988, Najran 11001, Saudi Arabia; 3 Department of Electrical Engineering, College of Engineering, Najran University, P.O. Box 1988, Najran 11001, Saudi Arabia; 4 Department of Textile Engineering, National Textile University, Faisalabad 37610, Pakistan; 5 U.S.-PAKISTAN Center for Advanced Studies in Energy (USPCASE), National University of Science and Technology, Islamabad 44000, Pakistan; 6 Department of Chemistry, Faculty of Science and Arts, Najran University, P.O. Box 1988, Najran 11001, Saudi Arabia; 7 Department of Surgery, College of Medicine, Najran University, P.O. Box 1988, Najran 11001, Saudi Arabia; 8 Department of Chemistry, Faculty of Science and Arts, Sharurah, Najran University, Sharurah 68342, Saudi Arabia

**Keywords:** polyethylene oxide, ginger extract, antibacterial, dynamic light scattering, viscoelastic properties

## Abstract

Synthetic antibiotics have captured the market in recent years, but the side effects of these products are life-threatening. In recent times, researchers have focused their research on natural-based products such as natural herbal oils, which are eco-friendly, biocompatible, biodegradable, and antibacterial. In this study, polyethylene oxide (PEO) and aqueous ginger extract (GE) were electrospun to produce novel antibacterial nanomembrane sheets as a function of PEO and GE concentrations. A GE average particle size of 91.16 nm was achieved with an extensive filtration process, inferring their incorporation in the PEO nanofibres. The presence of the GE was confirmed by Fourier transform infrared spectroscopy (FTIR) through peaks of phenol and aromatic groups. The viscoelastic properties of PEO/GE solutions were analysed in terms of PEO and GE concentrations. Increasing PEO and GE concentrations increased the solution’s viscosity. The dynamic viscosity of 3% was not changed with increasing shear rate, indicating Newtonian fluid behaviour. The dynamic viscosity of 4 and 5 wt% PEO/GE solutions containing 10% GE increased exponentially compared to 3 wt%. In addition, the shear thinning behaviour was observed over a frequency range of 0.05 to 100 rad/s. Scanning Electron Microscopy (SEM) analysis also specified an increase in the nanofibre’s diameter with increasing PEO concentration, while SEM images displayed smooth morphology with beadless nanofibres at different PEO/GE concentrations. In addition, PEO/GE nanomembranes inhibited the growth of *Staphylococcus aureus,* as presented by qualitative antibacterial results. The extent of PEO/GE nanomembrane’s antibacterial activity was further investigated by the agar dilution method, which inhibited the 98.79% *Staphylococcus aureus* population at 30% GE concentration.

## 1. Introduction

In 2018, a study published by the European Centre for Disease Prevention and Control (ECDC) estimated that about 33,000 people die every year of infections as a direct consequence of antibiotic-resistant bacteria [1]. Innovative antibacterial agents are needed against ever-evolving bacteria as they develop resistance to existing antibacterial agents over time. In addition, metal-based nanoparticles have been popular antibacterial agents in the past decades. Although metal-based nanoparticles are effective against bacteria and pathogens, there are doubts about their potential risk of toxicity [2,3,4,5,6,7]. The use of plant extracts and essential oils for therapeutic purposes has a long history due to their bulk availability and better biocompatibility. Extracts of different parts of plants (roots, seeds, leaves, flowers, and peels) had been used even in the Stone Age. Even modern medicine contains a significant amount of plant extracts because such extracts are valuable, cost-effective, and environment-friendly solutions against bacteria [8,9,10,11,12,13].

Ginger (Zingiber officinale Roscoe) is the rhizome of the monocotyledons member of the zingiberaceae plant family. Ginger’s medical and biological potential has been tested and studied for a broad range of biological activities such as analgesic, antitumor, antifungal, antioxidant, anti-allergic, anti-inflammatory, and antimicrobial. The antimicrobial properties of the ginger oils, ginger extract, and oleoresins depend upon their chemical compositions. The composition is ultimately dependent on cultivation conditions, climate, and soil factors, all of which may change the chemical constitution of the ginger [14]. Previous studies revealed that the phenolic compounds, along with combinations of β-sesquiphellandrene, cis-caryophillene, zingiberene, α-farnesene, α- and β-bisabolene are responsible for the antibacterial properties of ginger essential oil, extracts, and oleoresins (eugenol, shogaols, zingerone, gingerdiols, gingerols, etc.). These phenolic compounds are protein denaturants and lead to swelling and rupture of the bacterial cell [15,16,17,18]. Extraction technique is one of the many factors that affect the quality and antibacterial properties of bioactive compounds [19,20]. Solvent extraction (ethanol, methanol, water, etc.), microwave assisted extraction, ultrasonic assisted extraction, botanical extraction, and soxhlet extraction are the most commonly used methods to obtain plant extracts. All these methods require solvents or treatments prior to the extraction [21,22,23]. Ginger extract and essential oils have been used in hydrogels, films, and composites as antibacterial, antioxidant, and antifouling agents in the pharmaceutical and food packaging industries [24,25,26,27,28]. However, ginger is rarely studied as a nanofibre or part of a nanofibre due to its complex and coarse structure and processing issues. The separation of ginger nanoparticles from extract through vacuum filtration has not been investigated in the literature. The vacuum filtration process is used as a post-treatment for the purification of extracts [29]. In this study, ginger nanoparticles were separated by a cost-efficient and simple setup of vacuum filtration without any solvent.

Nanofibres are fine threads having diameters in the range of 50–500 nm and are well-known for their unique properties such as a large surface area to volume ratio, lightweight, high porosity, and bioactivity. Such physical properties have made them favourites for numerous sensor, membrane, filter, tissue engineering, wound dressing, and protective clothing applications [30,31,32,33,34,35,36,37,38]. There are several methods to produce nanofibres, such as drawing techniques, phase separation, self-assembly, freeze-drying synthesis, electrospinning, and interfacial polymerization of nanofibres [39,40,41,42]. Electrospinning is a unique method of producing nanofibre-based nanomembranes. This is a simple technique due to its ease of use, process flexibility, and manageable setup for nanomembrane production. The nanofibre structure can be modified using many different designs based on the spinnerets’ type, shape, size, number, arrangement of needles, and spinning methods [43,44,45,46]. Single needle-based electrospinning is a traditional method that has now been replaced with other techniques to increase the throughput, improve the efficiency, and improve the quality of the electrospinning process [47,48,49,50,51,52].

Polyethylene oxide (PEO) is a synthetic, biodegradable polymer belonging to the thermoplastics family. In addition, PEO is a versatile polymer because it is hydrophilic and soluble in water. PEO is an FDA-approved nontoxic and non-immunogenic polymer, which makes it biocompatible and human-friendly [53]. These attributes and the easy spinnability of PEO are reasons for the widespread use of PEO nanofibres in the biomedical and pharmaceutical fields [54,55,56].

This study combines ginger and PEO to produce nanomembranes for potential applications as a wound dressing. The impact of polymer concentration and ginger content were rheologically investigated and correlated with the production of nanofibre-based membranes. Agar qualitative and quantitative tests confirmed the nano fibres’ antibacterial activity.

## 2. Materials & Methods

### 2.1. Materials

Polyethylene oxide (99% pure) of an average molecular weight of 1,000,000 g/mol was purchased from Sigma Aldrich, St. Louis, MO, USA. HPLC Grade Water was purchased from Daejung, Korea. Ginger was purchased from the local market of Faisalabad, Pakistan.

### 2.2. Methods

#### 2.2.1. Filtration of the Ginger Extract

**Figure 1:**
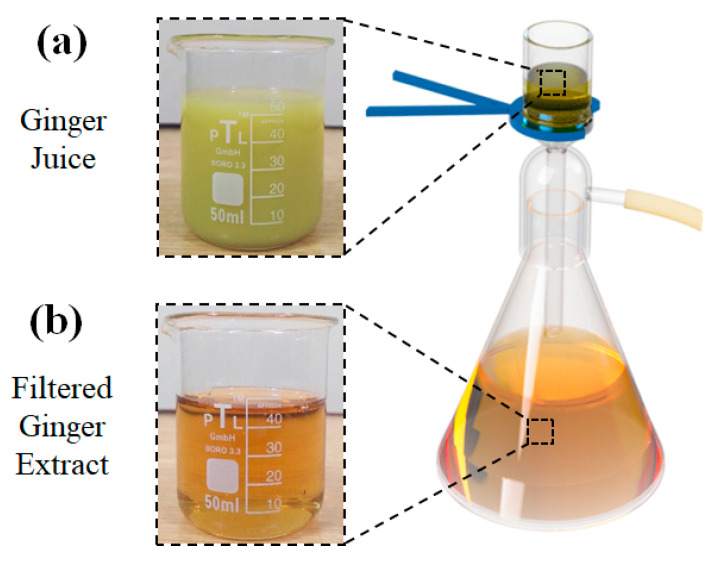
GE (**a**) before and (**b**) after filtration.

Ginger roots were thoroughly washed and dried at room temperature. After drying, the ginger was peeled and cut into small pieces. Ginger juice was extracted by passing ginger pieces through an electric juicer. Thick ginger juice was obtained, which was further purified using vacuum filtration, as shown in Figure 1. Ginger juice was passed through 0.45 µm and 0.22 µm pore-size filter papers consecutively to separate the coarser particles, and a transparent ginger extract was obtained, as shown in Figure 1b. Several trials were conducted to preserve the GE after filtration, and it was found that after 24 h, the GE slowly started to lose its transparency and became turbid in 2–3 days. GE was preserved at different temperatures, and sonication was used to avoid aggregation. It was observed that GE did not change in appearance and was reusable after sonication for almost a week when kept in an airtight bottle in a cold, dark place. However, the solutions were used within 12 h after filtration.

#### 2.2.2. PEO/GE Solution Preparation

A PEO stock solution was prepared by dissolving PEO powder in HPLC grade water at 80 °C. The polymer solution was continuously stirred for 4 h at 900 rpm by a magnetic stirrer to obtain a homogenous solution. Finally, the PEO stock solution was diluted to varying PEO and GE concentrations for the optimized electrospinning process, as shown in Table 1. Sample IDs and the description of the samples prepared for FTIR and Rheological testing is given in Table 2.

#### 2.2.3. Needleless Electrospinning

**Figure 2:**
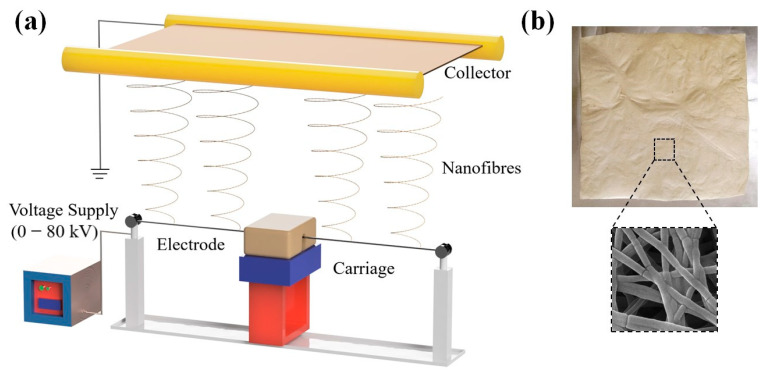
(**a**) Schematic illustration of the needless electrospinning process and (**b**) Photograph and SEM image of a PEO/GE nanomembrane.

An Elmarco needleless (NS) nano-spider was used to electrospin the PEO/GE nanomembranes. This setup has a movable carriage with a 50 mm capacity reservoir. Nanospider with wire electrodes has a closed feeding system to maintain a stable viscosity for hygroscopic or volatile solutions [57,58]. The schematic demonstration of this setup is shown in Figure 2a. The wire was soaked with PEO/GE solution in a closed carriage with a carriage speed of 50 mm/s. The distance between the electrode and collector was kept constant at 24 cm. As a result, continuous nanofibres were formed between 45 to 55 kV and deposited on aluminum foil. The schematic diagram, photographic, and SEM images are shown in Figure 2b.

## 3. Characterization

### 3.1. Dynamic Light Scattering

GE particle size analysis was conducted with a Zetasizer AT (Malvern, Kodaira, Japan). Almost 5~7 mm of GE was added to the glass cuvette with a round aperture and placed in the holder. All the samples were tested at 25 °C.

### 3.2. Frequency Sweep Test

A rotational Rheometer (AR1500ex) was employed to study the rheological behaviour of PEO/GE solutions at various PEO and GE concentrations. The frequency sweep test was conducted in the oscillatory shear mode at a rate of 0.05–200 rad/s at room temperature. The solution was placed on a 40 mm (diameter) plate with a 0.8 mm gap. The dynamic viscosity, storage modulus, loss modulus, and tan delta are then plotted against shear frequency.

### 3.3. Attenuated Total Reflectance-Fourier Transform Infrared Spectroscopy (ATR-FTIR)

Chemical analysis of PEO powder and PEO/GE nanofibres was carried out using the Bruker FTIR spectrometer (Alpha-E). The ATR-FTIR with Diamond (Platinum) crystal provides high quality spectral database for precise material verification and identification. IR spectra of PEO powder and PEO/GE nanofibres were scanned over a range of 500 to 4000 cm^−1^.

### 3.4. Field-Emission Scanning Electron Microscopy (FESEM)

A Hitachi S-3500-SEM was used to investigate the fibre morphology of PEO/GE nanofibres. Sputter coating of samples was done with gold particles. SEM images were taken at 20 keV with a magnification of 10,000×. The ImageJ software was used to measure the average diameter of the PEO/GE nanofibres from the SEM micrographs. An average of 100 fibres’ diameter was taken from each micrograph.

### 3.5. Agar Disc Diffusion Test

Agar disc diffusion and dilution tests were used for qualitative analysis of the PEO/GE nanofibres’ antibacterial resistance and ability to prevent bacterial growth. Muller-Hinton agar plates were prepared with tryptone soya agar (TSA), and the strains of cultured *Staphylococcus Aureus* were spread uniformly with a swab on the plate. PEO/GE nanofibre disks of 15 mm in diameter with different concentrations of ginger extract (10%, 20%, and 30%) were placed on prepared agar plates and then incubated at 37 °C for 24 h. After the incubation period, the plates were investigated for bacterial growth and the appearance of inhibition zones.

### 3.6. Agar Dilution Test

The agar dilution test was performed to quantify the antibacterial activity of PEO/GE nanofibres. *Staphylococcus aureus* was grown in a liquid agar medium. PEO/GE Extract nanofibres swatches were placed in the flasks with 50 mL nutrient agar, and samples were incubated in a shaker at 37 °C. After 24 h, solutions from flasks were placed on a nutrient agar plate and incubated at 37 °C for 24 h. The number of surviving bacterial colonies was counted immediately after inoculation and after 24 h incubation to calculate the percentage of bacterial reduction using the following formula.

$$\mathrm{Percentage}\;\mathrm{Reduction}=\frac{\left(A-B\right)}{A}\times 100\mathrm{CFU}\;(\mathrm{Colony}\;\mathrm{Forming}\;\mathrm{Units})$$

*A* = Number of bacteria recovered from the inoculated test sample immediately after contact.

*B* = Number of bacteria recovered from the inoculated test sample incubated over 24 h period.

## 4. Results & Discussion

### 4.1. Particle Size Analysis

**Figure 3:**
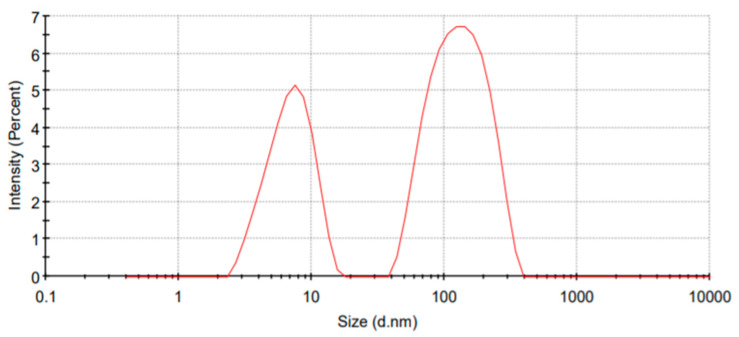
Size distribution analysis of GE.

In Figure 3, the size distribution graph of filtered GE indicates that the maximum nanoparticles lie between 5–300 d.nm. Almost 64.6% of particles have an average size of 140.2 d.nm, and 35.4% have 7.265 d.nm. Also, more than 25% of the 64.6% of larger particles have a size of less than 100 nm. These particles can be combined with nanofibres at the nanoscale. Also, the overall average particle size of GE is 91.16 d.nm which indicates its potential incorporation in nanofibres with a diameter greater than 150 nm. Particle size also affects antibacterial activity. Smaller particle sizes enhance the effect because of the large surface area and high rate of reactivity [59]. Although vacuum filtration had only been used as a post-treatment for purification of extracts, the results show it can be independently used as an extraction technique. It is cheap and straightforward, yet an effective and innovative method.

### 4.2. Chemical Group Analysis of Nanofibres

**Figure 4:**
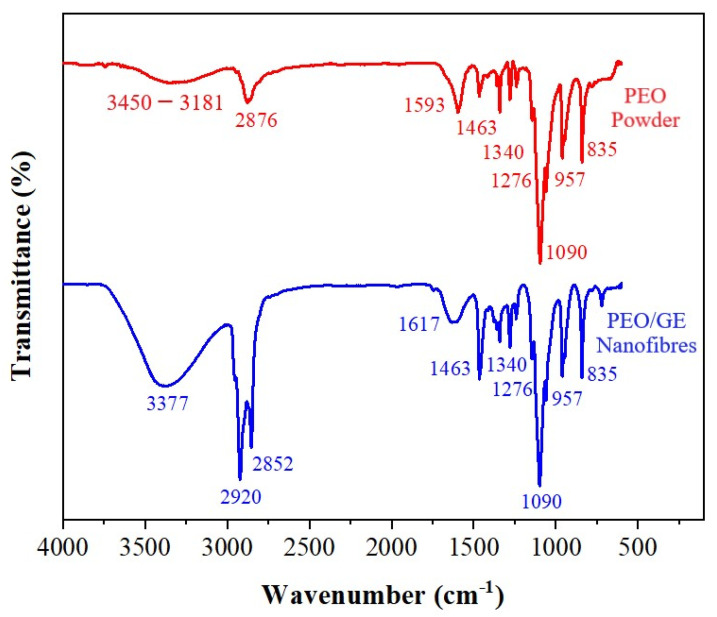
FTIR spectrum of PEO powder (red) and PEO/GE (blue) nanomembranes.

Figure 4 shows the FTIR spectra of PEO powder and PEO_5%_GE_30%_ nanofibres to examine the functional groups of GE and PEO. The band at 3450 to 3181 cm^−1^ in the red IR spectrum of PEO powder is due to the O-H stretching vibration. The peaks at 2876 cm^−1^, 1463 cm^−1^, and 1345 cm^−1^ can be assigned to C-H stretching of the methylene group and C-H bending, respectively. The relatively sharp peaks at 1090 cm^−1^, 957 cm^−1^, and 835 cm^−1^ correspond to C-O-C vibration and the asymmetric motion of CH_2_ [60,61].

In the blue curve, signifying PEO_5%_GE_30%_, there is the appearance of some visible peaks at 3377 cm^−1^, 2920 cm^−1^, 2852 cm^−1^, and 1617 cm^−1^. Carbohydrates (50–70%), lipids (3–8%), terpenes, and phenolic compounds are some of the major constituents of ginger. ginger’s phenolic compounds, including gingerol, paradols, and shogaol, are mainly responsible for the antibacterial activity of Ginger [62,63]. The broad peak at 3370 cm^−1^ corresponds to the O-H stretching and can be assigned to the formation of hydrogen bonds between PEO and these phenolic groups (e.g., 6-gingerol). The peaks at 2920 cm^−1^ and 2852 cm^−1^ represent the C-H and O-H stretching of the alkyl and carboxylic acid groups, respectively. The peak at 1617 cm^−1^ is attributed to the C=C stretching in the aromatic ring [64].

### 4.3. Rheological Properties

#### 4.3.1. Viscoelastic Behaviour of PEO/GE Solutions at Different PEO Concentrations

**Figure 5:**
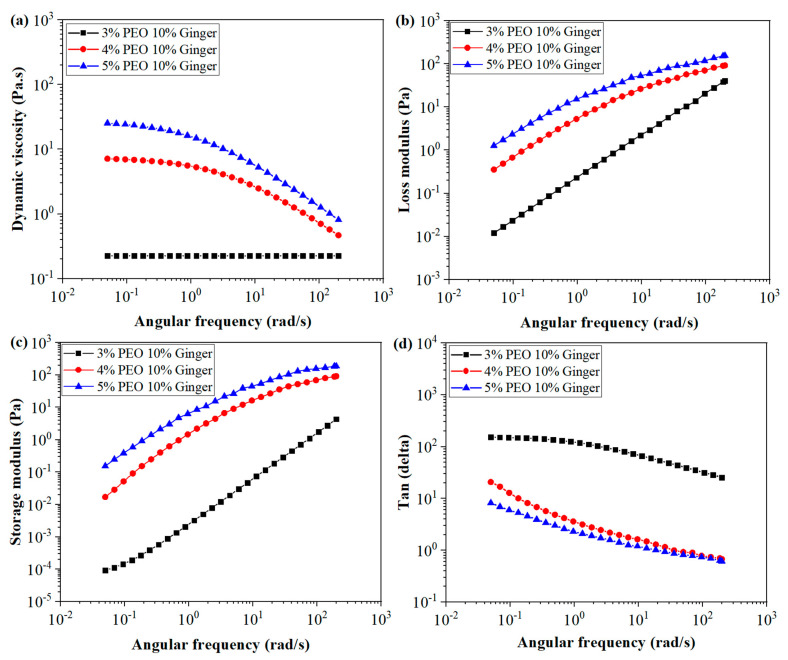
(**a**) Dynamic viscosity, (**b**) Loss modulus, (**c**) Storage modulus, and (**d**) Tangent delta of PEO/GE nanofibres (PEO_3%, 4%, 5%_ GE_10%_).

The viscoelastic properties of polymeric solutions depend on chain configuration, molecular weight, concentration, temperature, the nature of the solvent, and shear rate [65,66,67,68]. In this study, the concentration dependent viscoelastic behaviour of PEO/GE solutions was investigated by a frequency sweep test. It also has implications for solution electrospinnabilty and, ultimately, the morphology of nanofibres [69,70]. Figure 5 shows the dynamic viscosity, storage modulus, loss modulus, and tan delta of PEO/GE solutions with varying PEO concentrations while keeping the GE concentration constant at 10%. The viscosity of PEO_3%_ GE_10%_ remains constant as shear rate increases, exhibiting Newtonian behaviour. This trend is because of the low PEO_3%_ GE_10%_ concentration, where viscosity is associated with individual polymer chains. The PEO_4%_ GE_10%_ and PEO_5%_ GE_10%_ viscosities increase significantly with increasing PEO concentration, indicating that enough chains of PEO enable entanglement formation, leading to larger chain structures. This is because of the longer chain structure of PEO (higher molecular weight), which caused chain entanglements even at 4 wt%. Consequently, the viscosity increases exponentially with increasing concentration at 4 and 5 wt%. Both the PEO_4%_ GE_10%_ and PEO_5%_ GE_10%_ curves follow the shear thinning behaviour. In the first quarter, both solutions show a constant decrease in their dynamic viscosity because the chain disruption is low at lower shear rates and coils re-entangle instantaneously. The transition to shear thinning started at a lower frequency in the PEO_5%_ GE_10%_ solution because of higher chain entanglements than in PEO_4%_ GE_10%_. Then the shear thinning behaviour dominates with increasing shear rates because the rate of chain disruption exceeds the rate of re-coiling.

The dynamic viscoelastic parameters Loss Modulus G″, Storage Modulus G′, and Tangent Delta (Tanδ) for 3, 4, and 5 wt% PEO at room temperature are presented as a function of shear rate in Figure 5b–d, respectively. Storage modulus highlights the elastic part, while loss modulus indicates the viscous part of the non-Newtonian solution. Tangent delta, the ratio of loss to storage modulus, signifies the energy absorption behaviour of a material. Tangent delta values less than 1 imply that the material is inclined towards a viscous nature, while values higher than 1 indicate an elastic nature. The Tanδ of the PEO_3%_ GE_10%_ solution is significantly higher than 1 over the frequency range examined, which is an indication that the loss modulus of the solution is greater than that of the storage modulus. In other words, the viscous nature dominates over the elastic nature of a PEO_3%_ GE_10%_ solution. This is because of the lower concentration, where individual chains resist the change in their structure and result in the flow. However, as the shear rate increase, the Tanδ decreases, indicating that the PEO_3%_ GE_10%_ solution has reached the terminal region of viscoelasticity and exhibits dominated viscous behaviour. In the terminal region, rearrangements and slippage of polymer molecules relative to each other are observed. The PEO_3%_ GE_10%_ solution with the lowest PEO concentration has more space in the solvent to move freely with little chance of entanglements causing it to flow almost like a Newtonian fluid. PEO_4%_ GE_10%_ and PEO_5%_ GE_10%_ also have Tanδ over 1 at the start, and then both decrease with increasing shear rate. However, the Tanδ of both solutions has significantly lesser values than the PEO_3%_ GE_10%_ solution. Due to the higher PEO concentration, both G′ and G″ increases with increasing shear rate. However, the elastic modulus dominates with increasing shear rate, resulting in a lesser Tanδ value and reaching 1 around 30~40 rad/s for almost both solutions of PEO_4%_ GE_10%_ and PEO_5%_ GE_10%_. This is known as the gel point, where the loss and elastic modulus are equal. The elastic modulus dominates with increasing shear rates, and the Tanδ reduces from 1, indicating an elastic solution at higher shear rates. This can be explained based on a higher concentration of PEO with a longer chain structure, resulting in polymer chain interpenetration under the influence of shear force. The elastic behaviour of a solution depends on the formation of chain entanglements at different contacts that polymer coils make. Higher shear force promotes the chain entanglement points, resulting in increased elastic behaviour. Also, it is natural that a higher shear rate results in higher elastic responses.

#### 4.3.2. Viscoelastic Behaviour of PEO/GE Solutions at Different GE Concentrations

**Figure 6:**
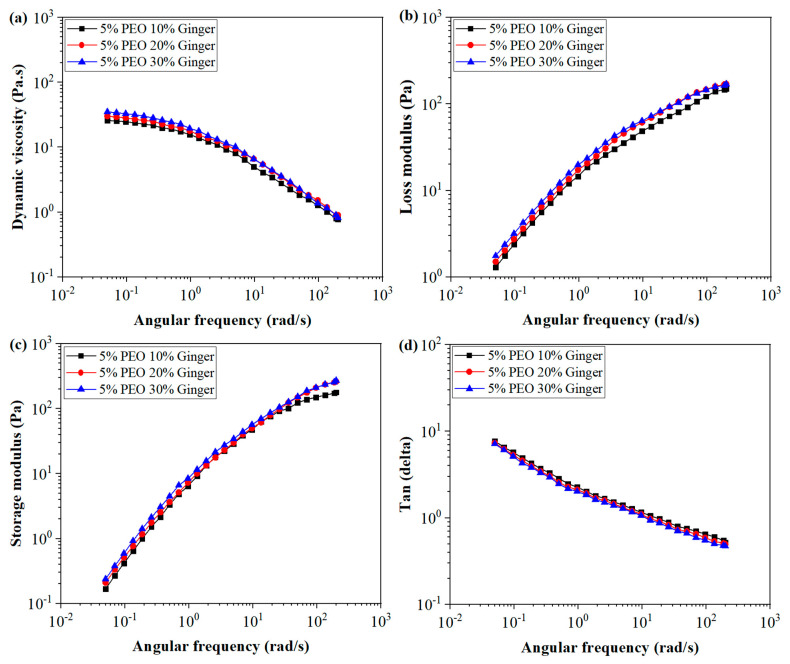
Dynamic viscosity, loss modulus, storage modulus, and tangent delta of PEO/GE nanofibres (PEO_5%_ GE_10%, 20%, 30%_).

**Figure 7:**
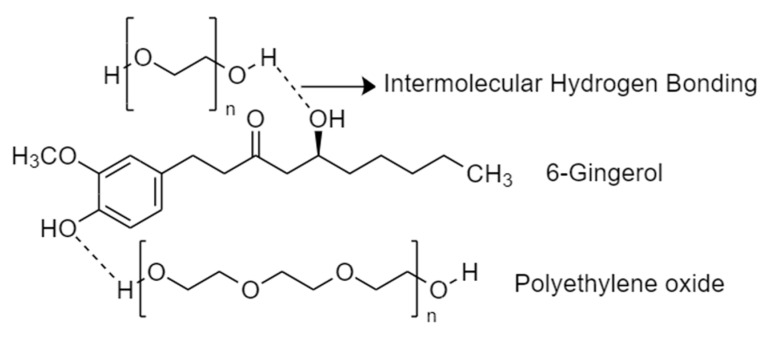
Schematic representation of hydrogen bonding between PEO and 6-Gingerol.

GE concentration can also affect the rheological behaviour of PEO/GE solutions, as rheological properties critically depend on the solvent polymer interactions. Water is a good solvent for PEO due to its higher polarity, and PEO molecules interact favourably with water. Dynamic viscosity, loss modulus, storage modulus, and Tanδ for solutions with a constant PEO concentration (5%) and varying GE concentration (10%, 20%, and 30%) are shown in Figure 6a–d. Increasing the GE concentration on solutions’ viscoelastic behaviour is not as drastic as varying PEO concentrations. However, the dynamic viscosity increases with increasing GE concentration in the solution. This can be explained by the polar interactions between PEO and active compounds of GE like 6-gingerol. Figure 7 shows the schematic diagram of intermolecular hydrogen bonding between PEO and 6-gingerol. Two possible reasons can explain the increasing viscosity behaviour. Firstly, a higher GE concentration might offer more hydrogen bonds to PEO, resulting in resisting flow and a higher viscosity. The second reason might be the larger size of 6-gingerol and other compound molecules in GE than the water molecules, resulting in swelling of the PEO chains with increasing GE concentration and higher viscosity. In addition, all three PEO/GE solutions follow shear thinning behaviour, indicating that the coil dimensions changed similarly for all solutions with varying GE concentrations. The Tanδ curve decreases with increasing GE concentration. These results align with the viscosity response, where more polymer and solvent interactions increase with GE concentration.

### 4.4. Nanofibre Morphological Analysis

**Figure 8:**
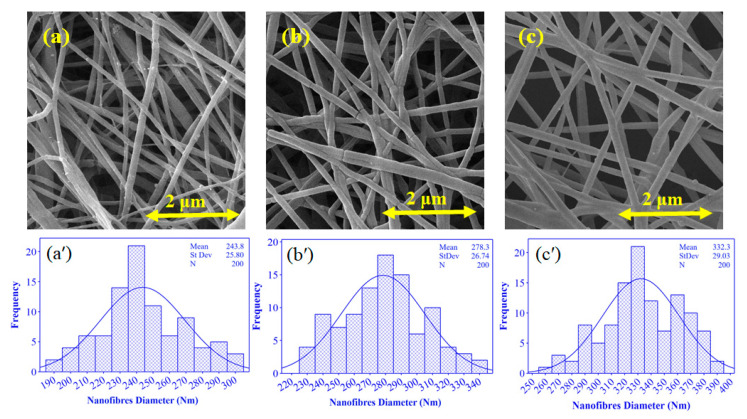
SEM images and histograms of (**a**,**a′**) PEO_3%_ GE_10%_, (**b**,**b′**) PEO_4%_ GE_10%_, and (**c**,**c′**) PEO_5%_ GE_10%_.

The morphology of nanofibres depends on the solution (solvent, molecular weight, concentration, viscosity, and temperature) and electrospinning process parameters (spinneret type and geometry, collector type, applied voltage, distance between collector, and spinneret) [71,72,73,74]. Figure 8 shows the SEM images and histograms of PEO/GE nanofibres electrospun at different PEO concentrations but under the same spinning conditions. SEM images indicate bead-less nanofibres with smooth morphology at all three PEO concentrations of 3, 4, and 5 wt%. Further, as PEO concentration increases, so does the average fibre diameter. The average nanofibre diameter of PEO_3%_GE_10%,_ PEO_4%_GE_10%,_ and PEO5_%_GE_10%_ is 242 nm, 269.3 nm, and 339.6 nm, respectively. This trend can be explained by increasing viscosity as PEO concentration increases. Solution viscosity has a direct impact on the nanofibres’ morphology. Large-diameter fibres form with a higher solution viscosity, and the diameter decreases with a reduced PEO concentration due to the reduced solution viscosity. As discussed previously, increasing the PEO concentration enhances chain entanglements, leading to higher viscosity. The chain entanglements form better elastic forces to resist stretching by electrostatic forces, resulting in nanofibres with a larger diameter and vice versa.

### 4.5. Qualitative Antibacterial Activity

**Figure 9:**
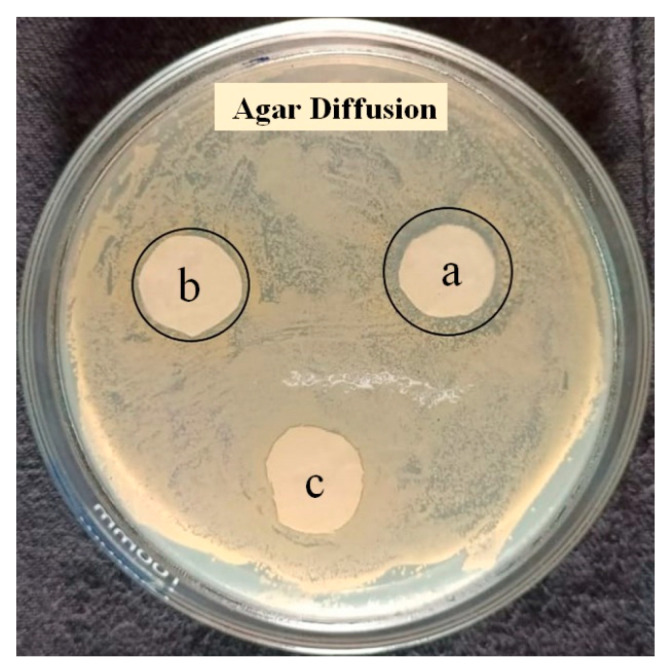
Inhibition zones of (a) PEO_5%_ GE_30%_, (b) PEO_5%_ GE_20%_, and (c) PEO_5%_ GE_10%_.

The Agar disc diffusion test of different polymer nanowebs was conducted to determine the GE antibacterial properties. Figure 9 shows the antibacterial property of PEO/GE nanofibres with varying GE concentrations on the growth of *Staphylococcus aureus*. Table 3 contains a description of sample ID, as well as their zone of inhibition and bacterial growth under specimen. The zone of inhibition is directly proportional to the GE concentration, as PEO/GE nanofibres with 30% GE have a 2 mm visible zone of inhibition. The other two solutions of, PEO_5%_ GE_20%_ and PEO_5%_ GE_10%_, have inhibition zones of 1.5 and 0.5 mm, respectively. Also, each PEO/GE sample inhibits bacterial growth, as there was no bacterial growth on the nanofibre discs.

### 4.6. Quantitative Antibacterial Activity

**Figure 10:**
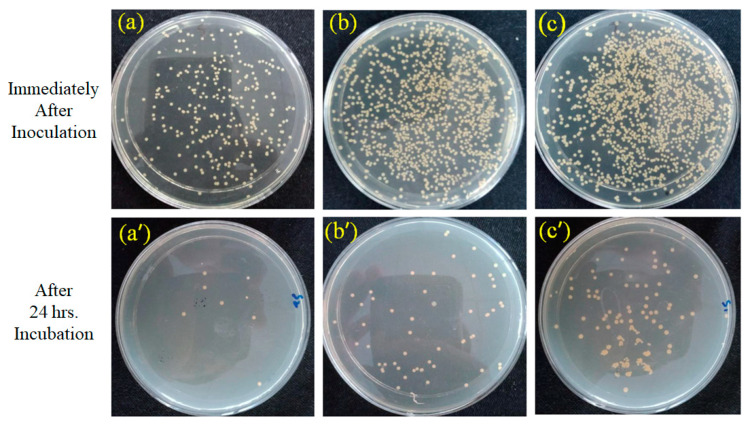
Bacterial colonies immediately after inoculation and after 24 h incubation of (**a**,**a′**) PEO_5%_ GE_30%_, (**b**,**b′**) PEO_5%_ GE_20%_, and (**c**,**c′**) PEO_5%_ GE_10%_.

The antibacterial activity of PEO/GE nanofibres was further evaluated quantitatively through the Agar dilution test. Figure 10 shows the reduction in bacterial colonies due to the bactericidal effect of PEO/GE before and after 24 h of incubation. The GE’s phenolic compounds and flavonoids kill bacteria by attacking the cell membrane and damaging the membrane potential, leading to protein leakage. The decrease in the protein content of bacteria causes bacterial cell death [75]. Table 4 shows the highest reduction (98.79%) of *Staphylococcus aureus* colonies in the PEO_5%_ GE_30%_ sample plate, and the PEO nanomembrane with 20% and 10% GE exhibit 96.03% and 94.47% reduction, respectively. These results support the Agar disc diffusion test, which found that the antibacterial activity of the PEO/GE nanomembrane increases with increasing GE concentrations.

## 5. Conclusions

Polyethylene oxide-based antibacterial nanowebs containing ginger extract were successfully fabricated as a function of PEO and GE concentrations. The effects of PEO and GE concentrations were investigated through rheological studies. The GE was obtained after vacuum filtration with an average particle size of 91.16 d.nm. The GE was incorporated in PEO solutions and fabricated by needleless electrospinning. Rheological analysis showed that the 3 wt% PEO showed Newtonian behaviour, as the viscosity did not change with shear rate. However, 4 and 5 wt% PEO showed shear thinning behaviour, resulting in Non-Newtonian fluids. Additionally, the dynamic viscosity increased with increasing the GE concentration. In addition, Tanδ decreased with increasing PEO concentrations and shear rates. Tanδ also reduced with increasing the GE concentration. However, the extent of Tanδ depreciation was larger with an increase in the PEO concentration than with increasing GE concentration. FTIR results validated the presence of GE in nanofibres as several peaks corresponding to GE chemical groups appeared in PEO/GE nanofibres. According to the SEM results, larger PEO/GE nanofibres with a higher PEO concentration produced fine nanofibres with a larger diameter (339.6 nm). In vitro agar diffusion and agar dilution results suggested promising antibacterial properties. The PEO nanofibres with 30% GE had a 2 mm inhibition zone and a reduction of 98.79%.

## Figures and Tables

**Table 1:** Design of the experiment (DOE).

Factor	Level (wt%)		
PEO conc. (wt%)	3	4	5
Ginger extract: HPLC water	30:70	20:80	10:90

**Table 2:** Sample ID description.

Sample ID	PEO Conc. (wt%)	Ginger Extract Conc. (wt%)
PEO_3%_ GE_10%_	3	10
PEO_4%_ GE_10%_	4	10
PEO_5%_ GE_10%_	5	10
PEO_5%_ GE_20%_	5	20
PEO_5%_ GE_30%_	5	30

**Table 3:** Zone of inhibition of the PEO/GE nanomembrane.

Sample	Sample Description	Zone of Inhibition (mm)	Bacterial Growth under Specimen
a	5% PEO 30% Ginger	2	Nil
b	5% PEO 20% Ginger	1.5	Nil
c	5% PEO 10% Ginger	0.5	Nil

**Table 4:** Bacterial reduction of the PEO/GE nanomembrane.

Sample	Sample Description	Reduction (%)
a	5% PEO 30% GE	98.79
b	5% PEO 20% GE	96.03
c	5% PEO 10% GE	94.47
